# TMT-Based Quantitative Proteomics Analysis Reveals the Panoramic Pharmacological Molecular Mechanism of β-Elemonic Acid Inhibition of Colorectal Cancer

**DOI:** 10.3389/fphar.2022.830328

**Published:** 2022-02-15

**Authors:** Yong Xia, Jinfan Yang, Chao Li, Xiaopeng Hao, Huixia Fan, Yuyang Zhao, Jinfu Tang, Xiufu Wan, Sen Lian, Jian Yang

**Affiliations:** ^1^ Key Laboratory of Precision Oncology of Shandong Higher Education, Institute of Precision Medicine, Jining Medical University, Jining, China; ^2^ State Key Laboratory Breeding Base of Dao-di Herbs, National Resource Center for Chinese Materia Medica, China Academy of Chinese Medical Sciences, Beijng, China; ^3^ Department of Biochemistry and Molecular Biology, School of Basic Medical Sciences, Southern Medical University, Guangzhou, China

**Keywords:** β-elemonic acid, colorectal cancer, mitochondria, cell cycle, ferroptosis

## Abstract

Colorectal cancer (CRC) is one of the most common cancers worldwide but has limited available therapeutic methods; therefore, there is a need to develop highly efficient prevention and treatment strategies. Here, we investigated the anti-cancer activity of β-elemonic acid (EA) in CRC *in vitro* and *in vivo*. Our results showed that EA inhibited cell proliferation and migration in the CRC cell lines SW480 and HCT116. Moreover, EA significantly suppressed the growth of transplanted colorectal tumors in nude mice. Interestingly, high-throughput tandem mass tag (TMT)-based quantitative proteomics indicated that EA mainly targets tumor mitochondria and attenuates the translation of 54 mitochondrial ribosome proteins, many of which are discovered significantly upregulated in clinical CRC patients. More interestingly, EA at a low concentration (lower than 15 μg/ml) repressed the cell cycle by downregulating CDK1, CDK6, and CDC20, whereas at a high concentration (higher than 15 μg/ml), caused a non-apoptotic cell death—ferroptosis *via* downregulating ferritin (FTL) and upregulating transferrin (TF), ferroxidase (CP), and acyl-CoA synthetase long-chain family member 4 (ACSL4). This is the first report on the panoramic molecular mechanism of EA against CRC, which would make great contributions to developing a novel drug for colorectal cancer therapy.

## Introduction

Colorectal cancer (CRC) is the second most common cancer in women, the third in men, and the fourth most deadly malignancy worldwide (Perez-Escalante et al., 2018; Dekker et al., 2019). The incidence of CRC is high in many countries of Europe, North America, and Asia, increasing yearly due to population aging and unhealthy lifestyles (Levine and Zbuk 2019; Moons et al., 2020). The most common treatments for CRC are surgery, chemotherapy, radiation therapy, targeted therapy, and immunotherapy; combination chemotherapy is the primary adjuvant strategy for stage III patients (Piawah and Venook 2019; Kong et al., 2020). Despite the advances in CRC diagnosis and treatment, the prognosis remains poor, and the occurrence of side effects and toxic reactions seriously limit the clinical therapeutic applications of these therapies (Leimkuhler et al., 2019; Kong et al., 2020). Therefore, the development of innovative anti-cancer approaches is necessary to prevent and treat CRC.

Plant-derived small-molecule compounds, such as paclitaxel, podophyllotoxin, camptothecin, capsaicin, and catechin, have been widely studied and applied for cancer prevention and treatment (Rizwanullah et al., 2018; Soflaei et al., 2018; Ranjan et al., 2019; Newman and Cragg, 2020). β-Elemonic acid (EA) is a type of triterpene known for its anti-inflammatory and anti-cancer functions (Atta-ur et al., 2005; Zhang et al., 2019). EA was reported to induce apoptosis and cell death of lung cancer A549 cells by elevating reactive oxygen species (ROS) levels through glutathione depletion, Bcl-2 downregulation, Bax upregulation, and inhibition of the MAPK signaling pathway (Wu et al., 2016). EA is also known to suppress the growth of prostate cancer cells and trigger the apoptosis of castration-resistant prostate cancer cells by attenuating the JAK2/STAT3/MCL-1 and NF-κB signaling pathways (Bao et al., 2021). However, the activity of EA against CRC remains unknown.

In this study, we aimed to investigate the potential anti-cancer activity of EA in CRC *in vitro* and *in vivo*, as well as to elucidate the underlying molecular mechanisms. Using CCK8, colony formation, and EdU methods, we found that EA inhibited the proliferation and migration of CRC cells in a concentration- and time-dependent manner *in vitro*. Consistently, *in vivo* studies demonstrated that EA significantly repressed subcutaneous tumor-bearing colorectal tumors. Proteomics and gene ontology (GO) function annotation revealed that the most prominent biological processes mediated by EA were mitochondria-related bioprocesses, including translational termination, translational elongation, and gene expression. We found that EA was able to downregulate 54 mitochondrial ribosomal proteins and diminish the mitochondrial membrane potential, indicating that mitochondria might be the dominant targeting organelles. In addition, EA at a low concentration (lower than 15 μg/ml) mainly repressed cell cycle by decreasing cyclin dependent kinase 1 (CDK1), cyclin dependent kinase 6 (CDK6), and cell division cycle 20 (CDC20), whereas high concentration (higher than 15 μg/ml) EA led to ferroptosis by downregulating ferritin (FTL) and upregulating ferroxidase (CP), transferrin (TF), and acyl-CoA synthetase long-chain family member 4 (ACSL4). This study is the first to investigate the role of EA in colorectal cancer and reveal the underlying mechanisms. Overall, our results help better understand the pharmacological activity of EA in CRC and lay a solid foundation for developing new anti-cancer therapies.

## Materials and Methods

### Cell Culture and Activity Measurement

CRC lines SW480 and HCT116 purchased from American type culture collection (ATCC; Manassas, VA, United States) were cultured in Dulbecco’s Modified Eagle Medium (HyClone, Logan, UT, United States) supplemented with 10% fetal bovine serum and 1% penicillin/streptomycin antibiotics at 37°C with 5% CO_2_. CRC line HT29 was from ATCC and cultured in McCoy’s 5A Medium supplemented with 10% fetal bovine serum and 1% penicillin/streptomycin. The control colorectal cell line NCM460 was purchased from BeNa Culture Collection (BNCC, Beijing, China) and cultured in RPMI 1640 medium supplemented with 10% fetal bovine serum and 1% penicillin/streptomycin. When the cells reached a density of 45–50%, EA (MedChemExpress, Monmouth Junction, NJ, United States) was added to the medium at different concentrations. The EA was solved into DMSO (Solarbio life science, Beijing, China). The Cell Counting Kit-8 (CCK8; Beyotime, Shanghai, China) was used to detect the inhibitory effect of EA on the activity of SW480 and HCT116, according to the manufacturer’s instructions.

### Colony Formation

CRC cells were cultured in 12-well plates (Corning, ME, United States) at 37°C with 5% CO_2_ for 12 h until the cells adhered to the wall. Next, they were treated with 0–20 μg/ml of EA for 10 days. The cells were stained using crystal violet solution (Beyotime) according to the manufacturer’s instructions.

### Cell Migration Assay

Cell migration assay was performed to check the inhibitory effect of EA on migration of CRC cells. Cells were seeded at 40% confluence into 12-well plates around culture inserts (ibidi, Grafelfing, Germany) and incubated at 37°C with 5% CO_2_. After 12 h, the inserts were removed, and the suspension cells were washed with phosphate-buffered saline (PBS). Fresh medium supplemented with 0–20 μg/ml EA was added. After 24 h incubation period, the width of cells scratches was observed under a microscope (Nikon, eclipase Ti-S, Tokyo, Japan), and images were captured.

### DNA Synthesis Assay

DNA synthesis was examined using the EDU-594 Cell Proliferation Assay Kit (C0078L, Beyotime), according to the manufacturer’s instructions, and staining results were recorded under a fluorescence microscope (Nikon, Japan).

### Proteomics Assay

To investigate changes in protein levels, we used 6-plex tandem mass tags proteomic assay. The proteomic experiments contained two groups: vehicle (DMSO) and EA treatment group. The CRC SW480 cells were treated with 15 μg/ml EA (or DMSO) for 24 h. Each group included three independent samples. The proteomic profiling including enzymatic hydrolysis, labeling, mass spectrometry and bioinformatics analysis (including GO and KEGG enrichment assay) was performed by Applied Protein Technology (Shanghai, China).

### Flow Cytometry for Cell Cycle and Apoptosis

For cell cycle detection, cells were fixed with 70% cold ethanol for 1 h and then treated with TritonX-100 containing RNaseA and propidium iodide (PI) for 20 min. The stained cells were detected on a flow cytometry (Beckman, cytoflex, United States). For apoptosis detection, cells were harvested with trypsin without Ethylenediamine tetraacetic acid (EDTA) and stained using the Annexin V-Fluorescein isothiocyanate (FITC)/PI double staining kit (Beyotime). The stained cells in both assays were detected using flow cytometry (Beckman, cytoflex, CA, United States).

### Xenografts

The mice experiment was approved by the ethics committee of Jining medical university, China. Female BALB/c nude mice were subcutaneously injected with 5 × 10^6^ SW480 cells and randomly divided into two groups (*n* = 5 in each group). When the tumor reached a volume of 200 mm^3^, the mice were intraperitoneally injected with 10 mg/kg of EA (treatment group) or DMSO (vehicle) every 2 days for 24 days. All mice were euthanatized with CO_2_ at 24 days post-injection, and their tumors, kidneys, livers, and hearts were placed in 10% formalin for fixation.

### Hematoxylin and Eosin, Immunochemistry, and Immunofluorescence Staining

Mouse tissues were fixed with 10% formalin, dehydrated, and embedded into paraffin blocks. The paraffin-embedded specimens were sectioned at a thickness of 4 μm using a microtome (Leica, RM2235, United States). The sections were dewaxed and stained with H&E. After being dewaxed and antigen repaired, the sections were stained for IHC and IF staining. The primary antibodies used in IHC were Rabbit-anti Ki67 (1:300; Abcam, Cambridge, United States) and Rabbit-anti FTL (1:100; Proteintech, China). The secondary antibodies and 3, 3′-diaminobenzidine were purchased from Boster Bio (Pleasanton, CA, United States). In IF staining, Rabbit-LC3 (1:250, Proteintech) was used as the primary antibody, and Goat-anti rabbit IgG H&L (Alexa Fluor 488, 1:400; Abcam) was used as the secondary antibody.

### Detection of Mitochondrial Membrane Potential

The effect of EA on the mitochondria of SW480 cells was detected using the Mitochondrial Membrane Potential Assay Kit (Beyotime) according to the manufacturer’s instruction. The stained cells were observed under an inverted fluorescence microscope (Nikon, Japan).

### Detection of ROS by H2DCFDA

H2DCFDA (MCE, Romulu, MI, United States), a cell-permeable probe, was used to detect changes in intracellular ROS induced by EA. SW480 cells in the EA treatment group and control were incubated with H2DCFDA at 37°C with 5% CO_2_ for 30 min, digested with trypsin, and suspended in PBS. Changes in ROS were detected using flow cytometry (Beckman, cytoflex, United States).

### Detection of Intracellular Iron

1×10^7^ SW480 cells (with or without EA treatment) were lysed by 1,000 μL lysis buffer. The concentration of intracellular iron was examined by a commercial available kit (Intracellular iron colorimetric assay kit) (E1042, Applygen, Beijing, China) according to the manufacturer’s instructions.

### Statistical Analysis

Data are presented as means ± standard deviation. Differences between two groups were identified using the independent-sample *t*-test, whereas those among more than two groups were identified using one-way ANOVA. Statistical significance was set at *p* < 0.05. All analyses were performed using SPSS 17.0 (IBM, Armonk, NY, United States).

## Results

### β-Elemonic Acid Inhibited Colorectal Cancer Cell Proliferation *in vitro*

**FIGURE 1:**
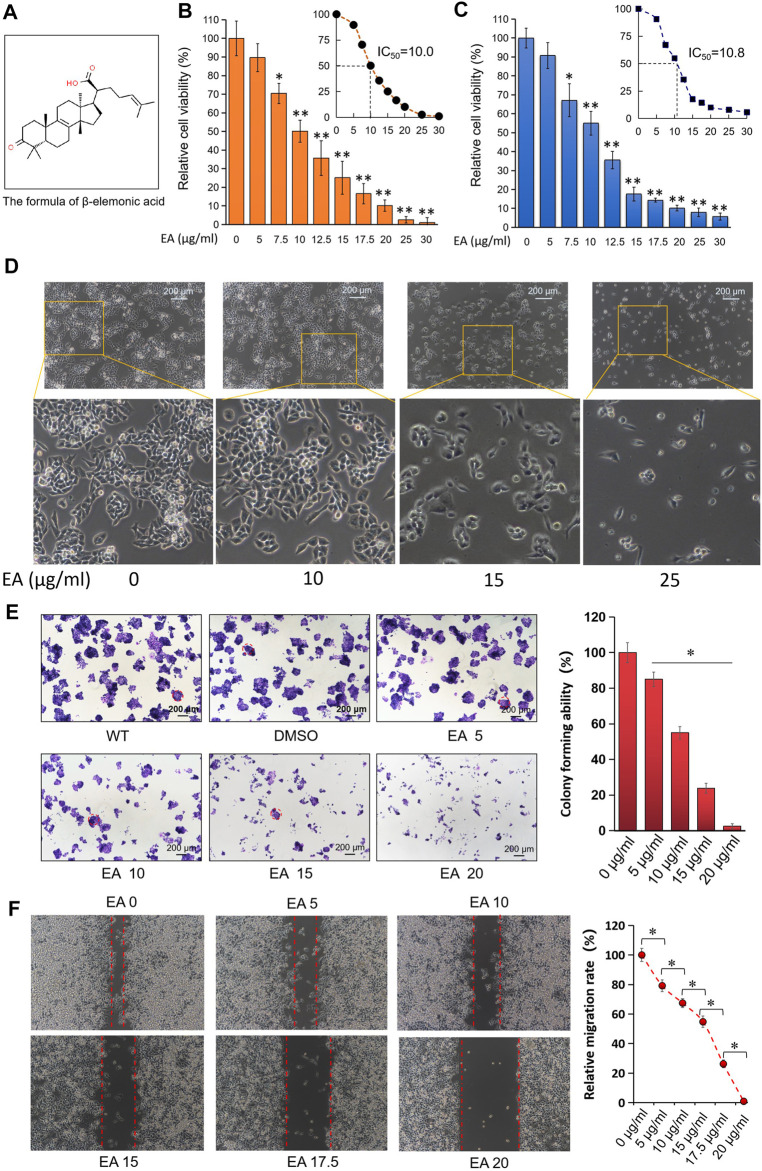
EA inhibited colorectal cancer cells in dose-dependent manner *in vitro*. **(A)** The formula of β-elemonic acid (EA); **(B)** Colorectal cancer SW480 cells were co-incubated in different concentrations of EA for 48h, and the cell activity was detected by the CCK8 kit. Four independent experiments were performed. The difference was calculated by a two-way *t*-test, *p < 0.05, **p < 0.01. **(C)** Colorectal cancer HCT116 cells were co-incubated in different concentrations of EA for 48 h, and the cell activity was detected by the CCK8 kit. Four independent experiments were performed. The difference was calculated by a two-way *t*-test, *p < 0.05, **p < 0.01. **(D)** SW480 cells were treated by EA for 48 h. And the EA-caused morphological changes in SW480 cells were photographed with an inverted microscopy. **(E)** 6 × 10^2^ SW480 cells were cultured into 12-well plates, and the cells were treated with different concentrations of EA for 10 days. The cell colonies were stained with crystal violet. The colonies with a diameter up to 200 μm were involved into the statistical comparison (see right column figure). The difference was calculated using a one-way ANOVA analysis, *p < 0.05. **(F)** The inhibitory effect of EA on the migration of colorectal cancer cells was examined by scratch assay. SW480 cells were co-incubated with 0–20 μg/ml EA for 24 h, and then the scratch width was measured. The scratch width was measured by ImageJ software. The rate of closure of cell scratches was quantified and shown in the right inset. Three independent experiments were performed. The difference was calculated by a two-way *t*-test, *p < 0.05.

Figure 1A showed the chemical structural formula of EA. The CCK8 assay showed that EA repressed the proliferation of CRC cells SW480 (half-maximal inhibitory concentration IC_50_, 10.0 μg/ml), HCT116 (IC50, 10.8 μg/ml) and HT29 (IC_50_, 12.1 μg/ml) in a concentration-depended and time-dependent manner; however, the normal colorectal cell line NCM460 was less sensitive to EA (IC_50_ of EA is 18.8 μg/ml) (Figures 1B,C, Supplementary Figures S1, S2). The IC_50_ difference laid the foundation for distinguishing cytotoxicity between normal colorectal cells and tumor cells. Due to the obvious cytotoxicities difference between colorectal cancer cells and normal cells, there could be potential for developing a new anticancer drug in the future. The morphological changes of SW480 cells in the EA treatment group are shown in Figure 1D. It was discovered that the cell morphology changed with the increase of EA concentration: when the EA concentration is higher than 15 μg/ml, the SW480 cells become insufficiently extended, and when the EA concentration reaches 25 μg/ml, the cells become round. Additionally, EA also suppressed colony formation of SW480 cells in a concentration-dependent manner (Figure 1E). The wound-healing assay revealed that EA inhibited the migration of SW480 cells in a concentration-depended manner and completely blocked their wound-healing capability at 20 μg/ml (Figure 1F).

### β-Elemonic Acid Inhibited Colorectal Tumor Growth *in vivo*

**FIGURE 2:**
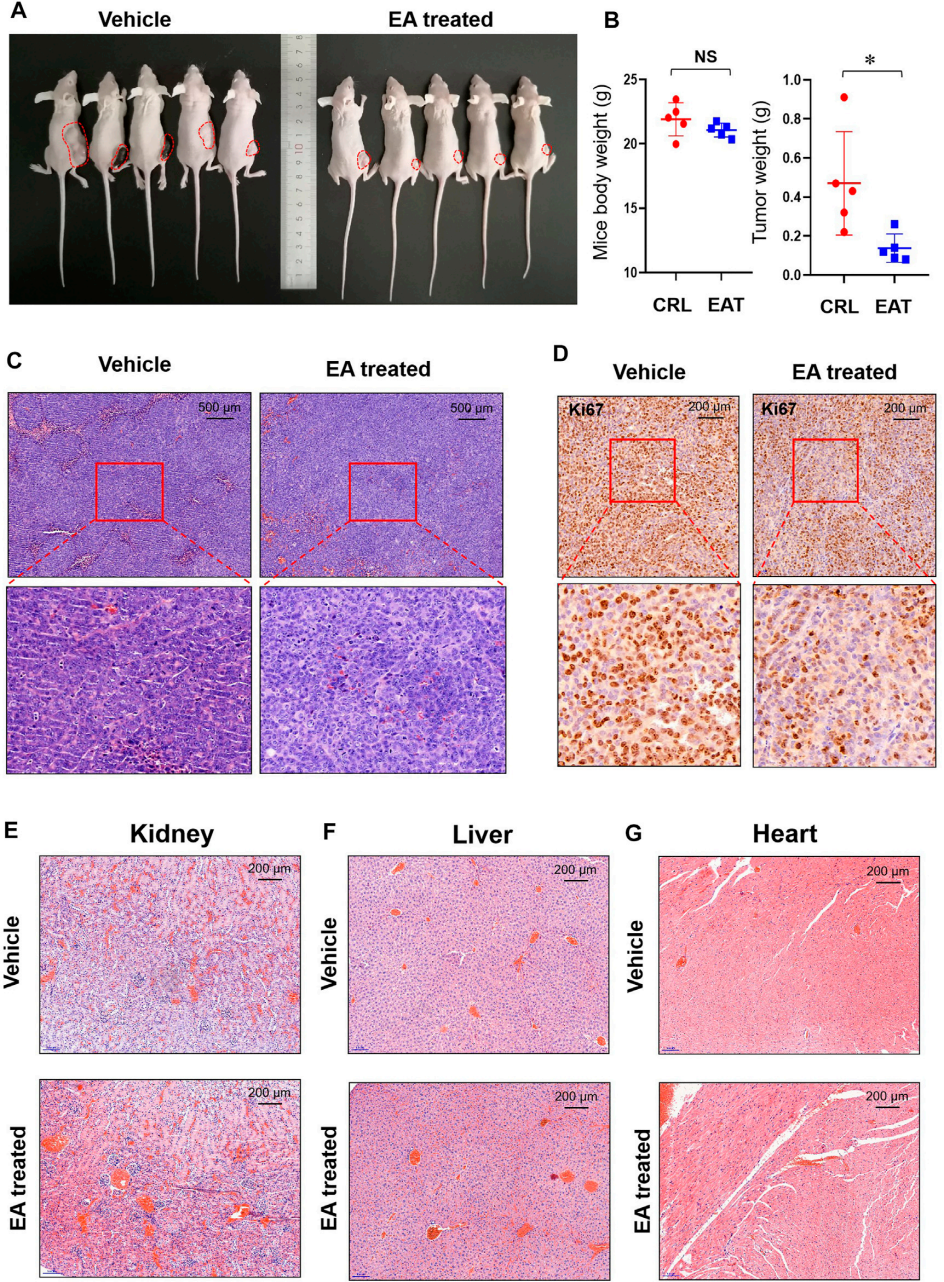
EA repressed colorectal tumor growth *in vivo*. **(A)** After experimental mice were sacrificed, the xenograft tumors (marked with red dotted lines) were photographed *in vivo*. **(B)** The tumor weight and mice body weight were measured and recorded. The difference was calculated by a two-way *t*-test, *p < 0.05, NS means no significant difference. **(C)** The EA-treatment xenograft tumors and control xenograft tumors were stained using H&E. **(D)** Immunohistochemistry (IHC) was used to detect Ki67 in both EA-treatment group and control group, and the stained slides were photographed by a microscope. **(E)** The mice kidneys in EA-treatment group and control group were stained with H&E dye. **(F)** The mice livers in EA-treatment group and control group were stained with H&E dye. **(G)** The mice hearts in EA-treatment group and control group were stained with H&E dye.

Assessment of xenograft models showed that EA treated subcutaneous tumors were obviously small than the DMSO treated subcutaneous tumors (Figure 2A). Figure 2B showed that the bodyweight of mice did not differ significantly between the EA treatment group and control; however, the tumor weight was significantly lower in the former than in the latter. Although the H&E staining result did not show obviously difference between EA treatment and control group (Figure 2C), IHC staining revealed that Ki67 levels were decreased in the EA treatment group compared with those in the control (Figure 2D). Furthermore, H&E staining did not reveal any differences between the kidney, liver, or heart sections of the EA treatment group and control, indicating the low toxicity of EA to mice (Figures 2E–G).

### Proteomic Analysis of the β-Elemonic Acid Anti-tumor Mechanism

**FIGURE 3:**
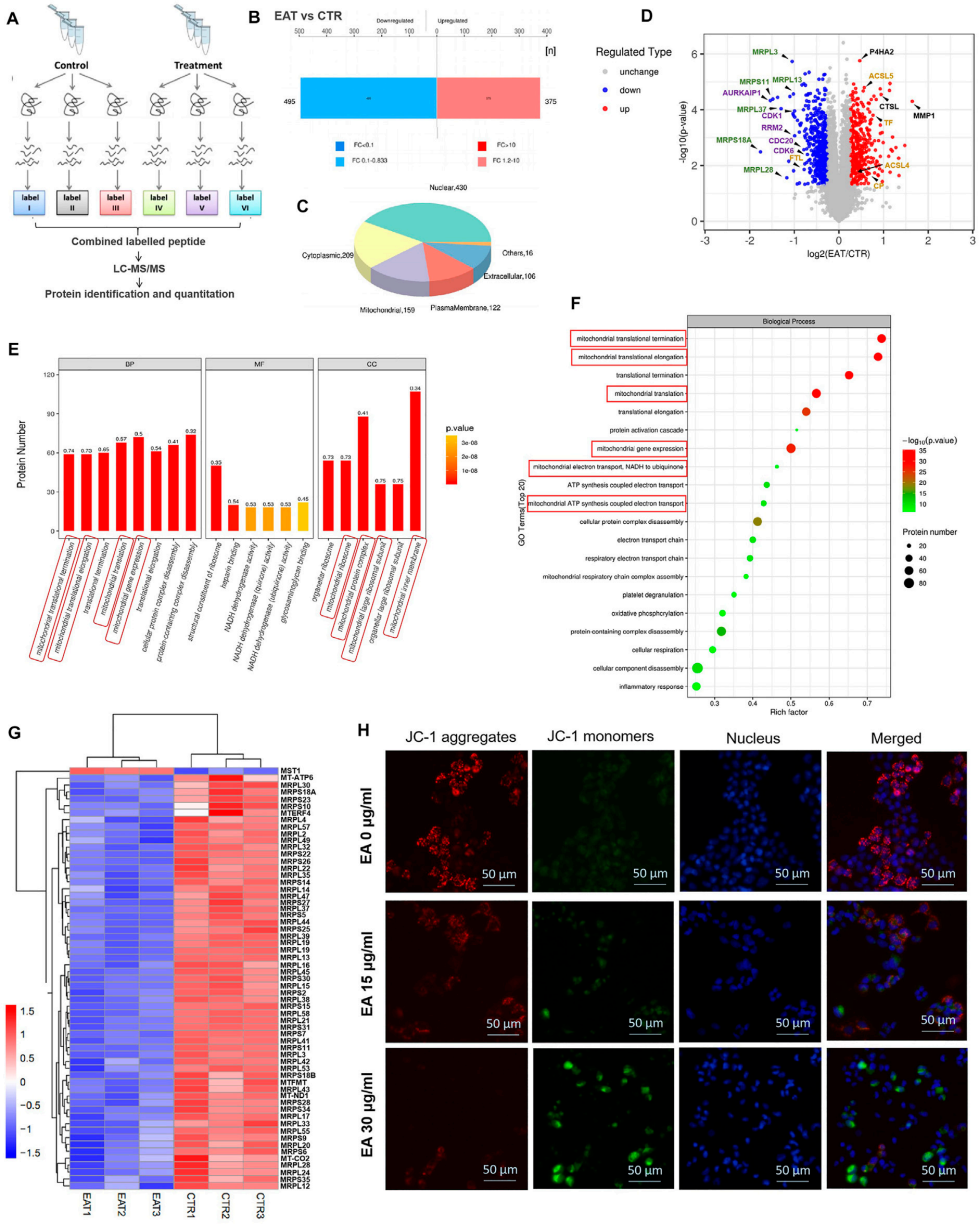
The proteomics result indicated the main target molecule of EA was mitochondrial-associated protein. **(A)** A schematic diagram of the process of proteomic analysis in this study. **(B)** Compared with control group, 495 proteins decreased and 375 proteins increased in the EA-treated group. **(C)** Sub-cellular localization of EA-induced proteins alteration. **(D)** Volcano plot of EA-induced proteins alteration. Red dots represent increased proteins, and blue dots represent decreased proteins. The green label was for mitochondrial ribosomal proteins, purple was for cell cycle-related proteins, yellow was for ferroptosis related proteins, and black was for examples of other proteins. **(E)** In the top 20 of enrichment analysis of the biological process, cellular component and molecular function, mitochondria-related pathways accounted for 40% (the mitochondria-related pathways were circled with red). The numbers above the bars indicate the Rich Factor. **(F)** In the top 20 pathways of GO enrichment analysis result, mitochondria-related pathways took the top spots in both rich factor and significance (the mitochondria-related pathways were circled red). **(G)** The EA-affected mitochondria-associated proteins were arranged and shown in the heat map. All the mitochondrial-related proteins (most of them are mitochondrial ribosomal proteins) were down-regulated by EA (MST1 is a non-mitochondrial protein, as a control). **(H)** The impact of EA on the mitochondrial membrane potential of colorectal cancer was detected by JC-1. When the mitochondrial membrane potential was high, JC-1 aggregated in the matrix of mitochondria, forming a polymer (J-Aggregates), producing red fluorescence; when the mitochondrial membrane potential was low, JC-1 could not gather in the matrix of the mitochondria, kept monomer type, producing green fluorescence.

The mass spectrometry proteomics data have been deposited to the ProteomeXchange Consortium (http://proteomecentral.proteomexchange.org) *via* the iProX partner repository with the dataset identifier PXD027833. Our proteomics data identified differences in protein expression between the EA treatment group and the control (Figure 3A). Using a fold change >1.2 (or <0.83) and *p* < 0.05 as filtering criteria, we discovered 870 differential proteins in the EA treatment group (Figure 3B); of these, 430 were located in the nucleus, 209 in the cytoplasm, 159 in the mitochondria, 122 in the cell membrane, and 106 in the extracellular matrix (Figure 3C). A volcano plot showed that mitochondrial ribosomal proteins (i.e., MRPL3, MRPS11, MRPL13, MRPL37, MRPS18A, and MRPL28) and cell cycle-related proteins (i.e., CDK1, CDK6, CDC20, AURKAIP1, and RRM2) were downregulated in the EA treatment group (Figures 3D,E). GO function annotation revealed that genes representing mitochondria protein-related pathways (translation termination and elongation) and biological processes were significantly enriched (Figures 3F,G). Additionally, many mitochondrial ribosomal proteins were highly expressed in CRC tissues but not in normal ones, indicating that they might be the dominant targets of EA (Supplementary Figure S3). Moreover, the mitochondrial membrane potential assay result showed a significant decrease in the mitochondrial membrane potential of SW480 cells in the EA treatment group (Figure 3H). Overall, EA repressed CRC cell proliferation by attenuating the translation of mitochondrial ribosomal proteins.

### β-Elemonic Acid Suppressed Colorectal Cancer Cell Cycle

**FIGURE 4:**
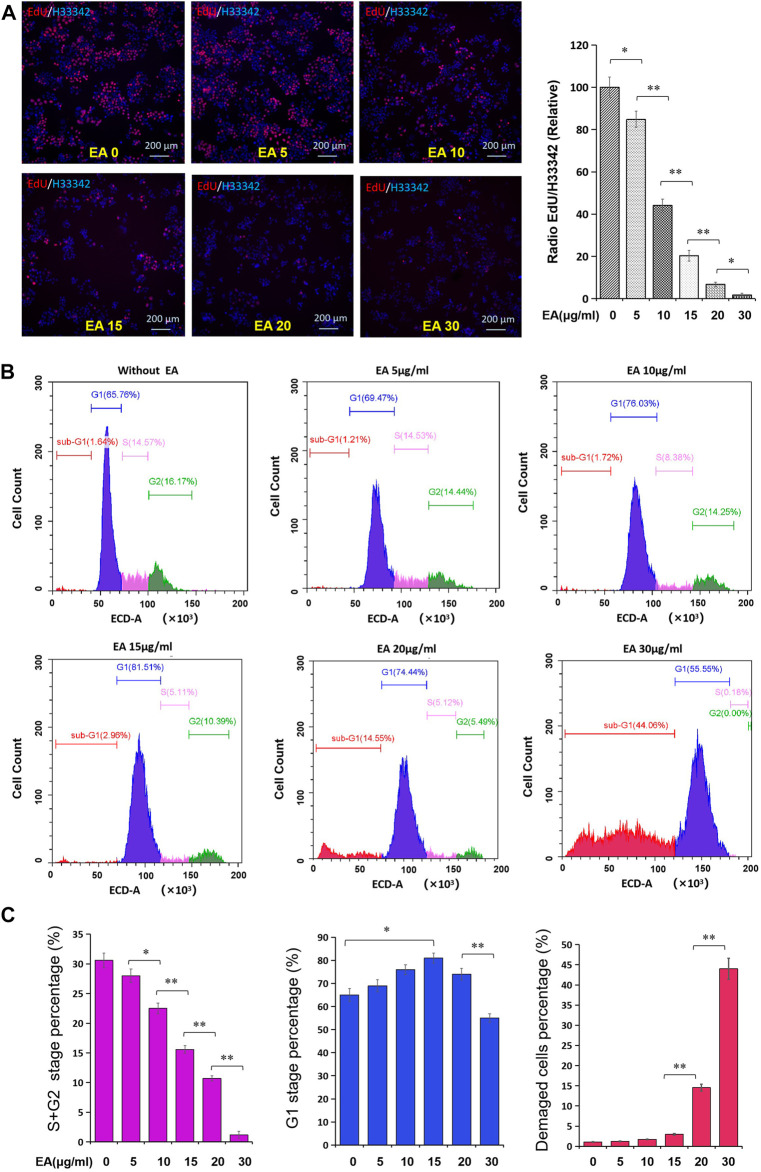
EA attenuated cell cycle of colorectal cancer cells. **(A)** The impact of EA on DNA synthesis of colorectal cancer cells was tested by the EdU kit. EdU positive stained cells were shown in red, and the cell nucleus stained by Hoechst33342 were shown in blue. The inset showed a quantitative calculation of the inhibitory effect of EA on DNA synthesis. The difference was calculated by a two-way *t*-test, *p < 0.05, **p < 0.01. **(B)** The impact of EA on cell cycle was investigated by flow cytometry with PI staining. Blue region represented the cells in the G1 phase, rose-pink region represented the cells in S phase, green region represented the cells in the G2 phase, and red region represented the damaged or defective cells. **(C)** The percentage of the S and G2 stage was quantitatively shown in rose-pink column figure, the percentage of the G1 phase was shown in blue column, and the percentage of damaged cells was shown in red column.

On examining DNA synthesis, we found that EA significantly inhibited DNA synthesis in and reduced the proliferation rate of SW480 cells in a concentration-dependent manner (Figure 4A). Flow cytometry revealed that EA significantly repressed the S and G2 phases in a concentration-dependent manner but did not affect the G1 phase (Figures 4B,C). It is worth noting that EA at a concentration of 20 μg/ml significantly increased the percentage of cells in the sub-G1 phase, suggesting the induction of CRC cell death (Figures 4B,C).

### β-Elemonic Acid Induced Colorectal Cancer Non-apoptotic Cell Death

**FIGURE 5:**
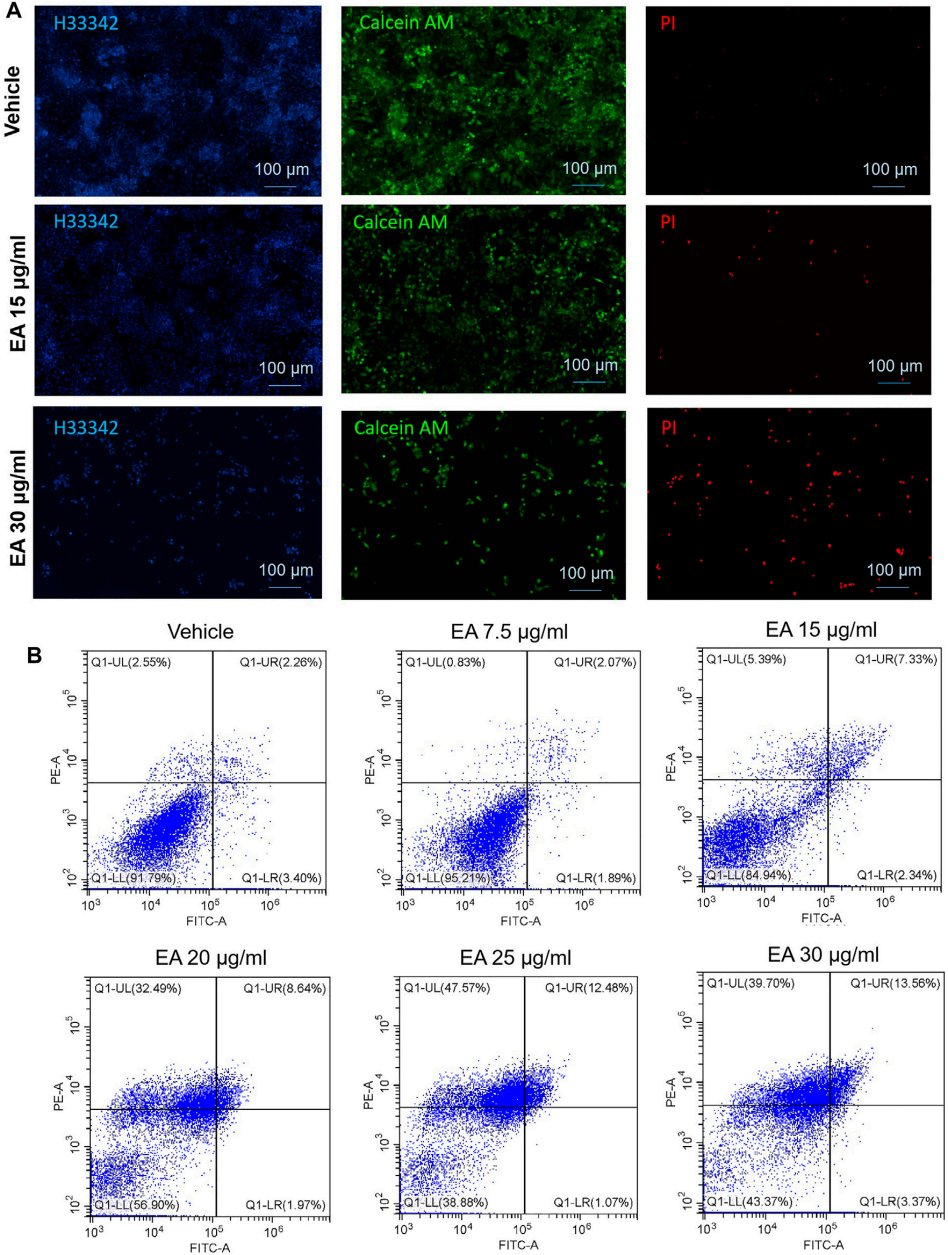
EA induced colorectal cancer cell death *via* a non-apoptotic manner. **(A)** Living cells were stained by calcein AM (green fluorescence), dead cells were stained by PI (red fluorescence), and all the cell nuclei were stained by Hoechst33342 (blue fluorescence). **(B)** PI and Annexin V double stained cells were analyzed by flow cytometry. The *X*-axis showed the FITC intensity representing the Annexin V on the cell outer membrane, and the *Y*-axis represented the PI staining intensity of cells. In the four quadrants, Q1-LR represented the early apoptotic cells population, and Q1-UR represented the late apoptotic cells population.

Calcein AM/PI double staining was employed to verify EA inducing CRC SW480 cell death. As shown in Figure 5A, 15 μg/ml led to a small number of cells dying, while 30 μg/ml caused a large number of cells to die (the dead cells were PI positive stained). Annexin V-FITC/PI double staining showed that EA induces cell death in a concentration-depended manner (Figure 5B). Flow cytometry revealed that EA increased fluorescence intensity of Annexin-V and PI, but not as Annexin-V positive/PI negative quadrant, suggesting non-apoptotic cell death.

### β-Elemonic Acid Induced Colorectal Cancer Cell Ferroptosis

**FIGURE 6:**
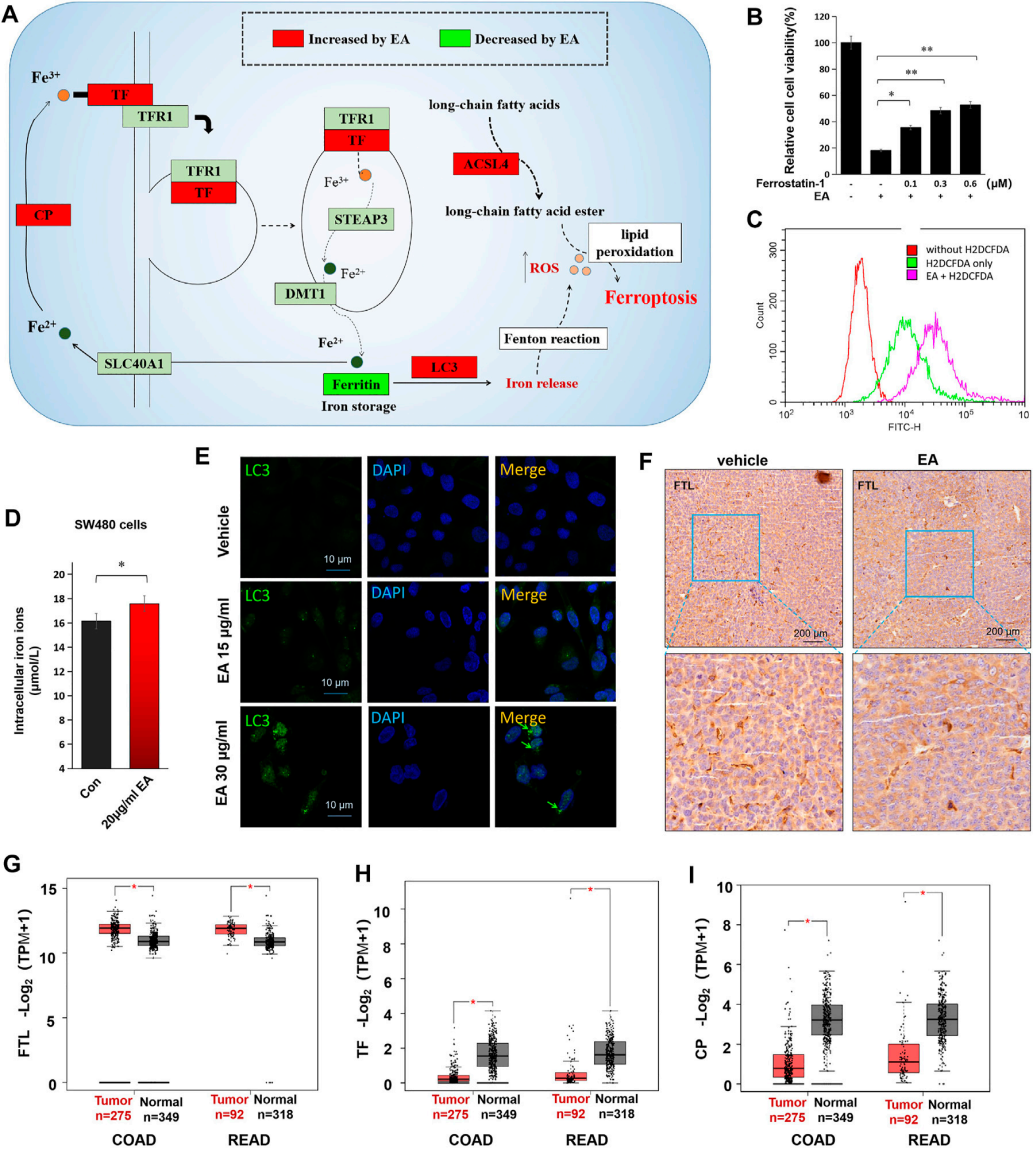
EA induced colorectal cancer cell death *via* triggering ferroptosis. **(A)** The alteration of ferroptosis-related proteins caused by EA treatment (the diagram was drawn according to proteomic results and ferroptosis KEGG map). **(B)** The rescue effects of ferrostatin-1 (an iron death inhibitor) on the 20 μg/ml EA-induced cell inactivation were detected by the CCK8 kit. The difference between two groups was calculated by a two-way *t*-test, *p < 0.05, **p < 0.01. **(C)** EA-induced intracellular ROS was tested by H2DCFDA. The red curve represented the background without the ROS probe, the green curve represented the ROS intensity of cells without EA treatment, and the rose-pink curve represented the ROS intensity of EA-treated cells. The EA concentration in this experiment was 20 μg/ml. **(D)** The alteration of intracellular iron concentrations of EA-treated SW480 cells. The difference between two groups was calculated by a two-way *t*-test, *p < 0.05. **(E)** The EA-elevated LC3 was verified by cell immunofluorescence experiment in SW480 cells. LC3 was stained by green fluorescence and the nucleus was stained by DAPI with blue fluorescence. **(F)** The EA-repressed ferritin (FTL) was examined by IHC in xenograft tumors. The FTL was stained with brown color, and the nucleus was stained by hematoxylin (purple). **(G–I)** The expression of FTL, TF and CP in clinical colorectal cancer tissue and normal tissue. Data were from the online platform GEPIA (http://gepia.cancer-pku.cn/index.html). COAD: Colon adenocarcinoma; READ: Rectum adenocarcinoma, *p < 0.05.

Kyoto Encyclopedia of Gene and Genomes (KEGG) enrichment analysis showed the downregulation of ferritin (FTL) and the upregulation of ferroxidase (CP), transferrin (TF), and acyl-CoA synthetase long-chain family member 4 (ACSL4) in the EA treatment group, suggesting EA might trigger ferroptosis (Figure 6A). The results were confirmed using ferrostatin-1, a ferroptosis inhibitor, which effectively rescued EA-repressed CRC cell activity partially (Figure 6B). Additionally, we found that EA markedly increased intracellular ROS in SW480 cells (Figure 6C), elevated the intracellular iron concentration (Figure 6D), altered and enhanced LC3 in IF stained SW480 cells, and reduced FTL in the xenograft model (Figures 6E,F). The major function of FTL is to store soluble and nontoxic iron, whereas LC3-mediated degradation of FTL leads to iron release. We also analyzed the expression of TF, CP, and FTL in CRC using the online big data platform (http://gepia.cancer-pku.cn/index.html). The levels of TF and CP were significantly lower in CRC tissues than in normal ones, whereas those of FTL were significantly higher (Figures 6G–I). Therefore, an increase in FTL levels and a decrease in TF and CP levels might be positively correlated with the occurrence of CRC, whereas EA ameliorates malignancy by downregulating FTL and upregulating TF and CP. Moreover, due to the upregulation of TF, the intracellular iron concentration increased (Figure 6D), which may contribute to promoting ferroptosis.

**FIGURE 7:**
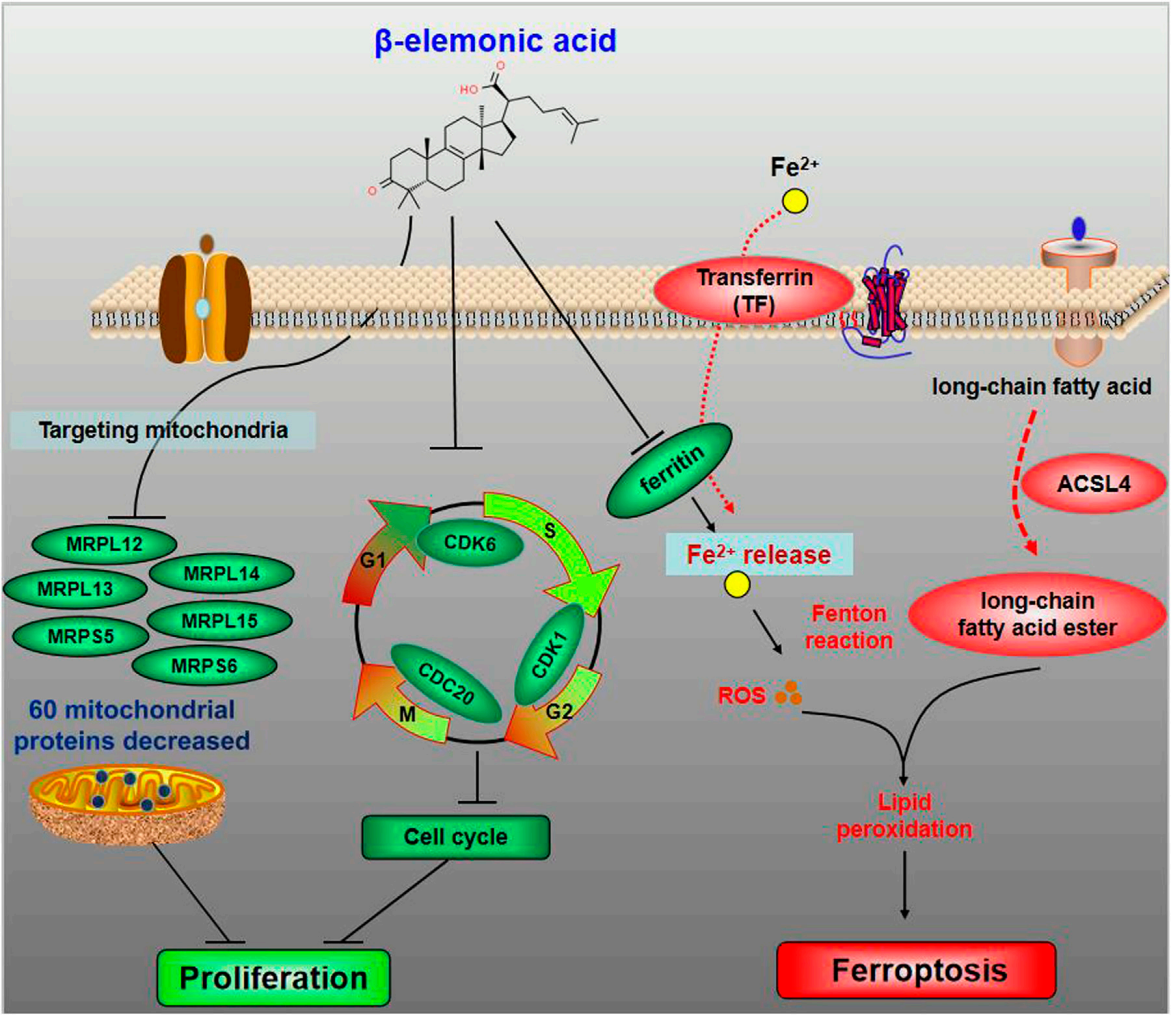
The schematic diagram for the molecular pharmacological mechanism of EA suppressing colorectal cancer. The dominant target organelle of EA for colorectal cancer is mitochondria. EA can reduce 60 mitochondrial proteins, among which more than 90% are mitochondrial ribosomal proteins. Moreover, based on what we discovered, the anticancer mechanism of EA could be described as follows: at low concentration, EA inhibited the cell cycle by downregulating CDK1, CDK6 and CDC20; at high concentrations, EA induced ferroptosis by upregulation of TF, CP and ACSL4, and down-regulation of FTL.

In summary, EA reduced 54 mitochondrial ribosomal proteins (e.g. MRPL12, MRPL13, MRPL14, MRPS5, etc.) in CRC SW480 cells (Figure 7). At low concentration (lower than 15 μg/ml) EA mainly inhibited the cell cycle by downregulating CDK1, CDK6 and CDC20; whereas at high concentrations (higher than 15 μg/ml), EA induced ferroptosis by upregulation of TF, CP and ACSL4, and down-regulation of FTL (Figure 7).

## Discussion

EA is a plant-derived compound with attractive bioactivity (Zhao et al., 2020). The anti-cancer properties of EA have been previously reported (Wu et al., 2016; Zhao et al., 2020); however, little is known about its pharmacological action and effects on CRC. Here, we revealed the inhibitory effect of EA in CRC *in vitro* and *in vivo* and investigated the underlying molecular mechanism combining high-throughput proteomics with traditional molecular techniques.

Our results showed that the expression of many mitochondrial ribosomal proteins was positively correlated with CRC occurrence, whereas some of them (i.e., MRPS5, MRPS6, MRPL3, MRPL12, MRPL13, and MRPL14) were downregulated by EA (Figure 3G). Thus, EA might be able to repress CRC by targeting mitochondrial proteins. It is known that mitochondrial ribosomal protein S5 (MRPS5) enhances the metabolic flexibility of liver cancer stem cells; however, the role of other mitochondrial ribosomal proteins in cancer pathogenesis remains unclear (Wei et al., 2019). Although previous studies have suggested that targeting mitochondria could be an effective anti-cancer strategy, this is the first report on a compound that affects the expression of mitochondrial ribosomal proteins, allowing for the development of novel therapeutic methods.

It has been previously reported that mitochondrial (mt) DNA mutations and mitochondrial metabolic abnormalities are closely related to tumorigenesis, and cancer cell mitochondria have a significantly higher transmembrane potential than normal cells (Chen 2012) (Wang et al., 2010). Besides being an essential organelle for energy metabolism, mitochondria also play an essential role in the cell death mechanism by acting as the site of ROS production (Zong et al., 2016). Previous studies showed that promoting mitochondrial ROS production to induce cancer cell death enhances chemotherapy (Yun et al., 2015). In the present study, EA significantly reduced the mitochondrial membrane potential of CRC cells and increased intracellular ROS, confirming its anti-cancer properties.

Compared with normal cells, tumor cells proliferate at a significantly higher rate, requiring more iron and producing more free radicals; therefore, they are more vulnerable to ferroptosis inducers (Hayes et al., 2020) (Hassannia et al., 2019). FTL is the major intracellular iron storage protein in eukaryotes, which stores iron in a soluble and non-toxic state (Bu et al., 2012). Its downregulation results in intracellular iron homeostasis imbalance, accelerating ferroptosis. Tang et al. reported that autophagy triggers ferroptosis by ferritin degradation and iron release (Hou et al., 2016). Here, we found that EA upregulated LC3, a marker of autophagy, and downregulated FTL, enhancing ferroptosis.

Iron enters the cell in the form of Fe(III) mediated by ferroxidase and transferrin. Ferroxidase (CP) plays a role in the peroxidation of Fe(II) to Fe(III) (Ebrahimi et al., 2012), and transferrin (TF) is responsible for transporting ferric iron into cells (Luck and Mason, 2012). We found that the expression of CP in CRC tissues was significantly lower than that in normal tissues, and these results are inconsistent with those reported for other cancers such as glioma (Manjula et al., 1992) and lung carcinoma (Matsuoka et al., 2018). Additionally, EA-induced CP increased the conversion of Fe(II) transferrin to Fe(III) transferrin, which enhanced the iron transportation into the cells. Thus, EA upregulated TF and CP, leading to excessive intracellular iron in CRC cells and, consequently, accelerating ferroptosis.

ACSL4 converts free long-chain fatty acids into fatty acyl-CoA esters, playing a pivotal role in lipid biosynthesis (Chen et al., 2021). Lipid peroxidation is the leading cause of ferroptosis, and thus, ACSL4 might be a direct or indirect promoter of ferroptosis. In recent years, many literatures have reported that ACSL4 can increase the cells sensitivity to ferroptosis (Doll et al., 2017) or directly enhance ferroptosis (Cheng et al., 2020). In the present study, EA upregulated ACSL4 and increased intracellular ROS, inducing CRC cell ferroptosis.

This article is the first to report the inhibitory effect of EA on colorectal cancer cells and its comprehensive pharmacological network mechanism. Targeting mitochondria and inducing ferroptosis are the two highlights of EA in cancer inhibition. Admittedly, there are still a few limitations in this study, e.g. the direct interaction proteins of EA have not been captured. In the following study, we will use the pulldown-mass spectrometry strategy to examine the direct interaction proteins of EA using biotin-conjugated EA to further clarify the molecular mechanism of EA.

Overall, EA is a potent anti-cancer compound that suppresses the translation of many mitochondrial ribosomal proteins and cell cycle proteins in a concentration-depended manner. Furthermore, EA at high concentration induces ferroptosis by downregulating FTL and upregulating TF, CP, and ACSL4. Thus, our study reveals the anti-cancer mechanism of EA at the cellular and molecular levels and provides important references for developing effective therapeutic strategies for CRC.

## Data Availability

The datasets presented in this study can be found in online repositories. The names of the repository/repositories and accession number(s) can be found below: http://proteomecentral.proteomexchange.org; PXD027833.
